# A pertinent approach to solve nonlinear fuzzy integro-differential equations

**DOI:** 10.1186/s40064-016-2045-4

**Published:** 2016-04-14

**Authors:** S. Narayanamoorthy, S. P. Sathiyapriya

**Affiliations:** Department of Mathematics, Bharathiar University, Coimbatore, TamilNadu 641046 India

**Keywords:** Fuzzy nonlinear integro-differential equations, Fuzzy functions, Homotopy perturbation method, Approximate solutions, Stability, Convergence

## Abstract

Fuzzy integro-differential equations is one of the important parts of fuzzy analysis theory that holds theoretical as well as applicable values in analytical dynamics and so an appropriate computational algorithm to solve them is in essence. In this article, we use parametric forms of fuzzy numbers and suggest an applicable approach for solving nonlinear fuzzy integro-differential equations using homotopy perturbation method. A clear and detailed description of the proposed method is provided. Our main objective is to illustrate that the construction of appropriate convex homotopy in a proper way leads to highly accurate solutions with less computational work. The efficiency of the approximation technique is expressed via stability and convergence analysis so as to guarantee the efficiency and performance of the methodology. Numerical examples are demonstrated to verify the convergence and it reveals the validity of the presented numerical technique. Numerical results are tabulated and examined by comparing the obtained approximate solutions with the known exact solutions. Graphical representations of the exact and acquired approximate fuzzy solutions clarify the accuracy of the approach.

## Background

At present time, the study of fuzzy integro-differential equations is an issue of remarkable consideration because it is one of the modern mathematical fields that arise from the modeling of uncertain physical, engineering and medical problems and are useful in studying the observability of dynamical control systems. The notion of fuzzy set theory has recently increased due to its adaptability and have been of great interest by several researchers. Concept of fuzzy numbers was originally introduced by Zadeh (1965) which led to the definition of fuzzy mappings and fuzzy control (Chang and Zadeh 1972). The basic arithmetic structure of fuzzy numbers was given by (Dubois and Prade 1978) and they observed fuzzy numbers as a collection of $\alpha$-levels, $0 \le \alpha \le 1$. In this regard various authors (Dubois and Prade 1982; Goetschel and Voxman 1986; Puri and Ralescu 1983, 1986; Seikkala 1987) made a significant contribution to fuzzy calculus and gave it a more applicable representation. Also few authors have predominantly worked on fuzzy integro-differential equations (Mosleh and Otadi 2012) and on its existence and uniqueness (Balasubramaniam and Muralisankar 2001; Abu Arqub et al. 2015). As these equations are typically complicated to solve analytically & so various authors focus on the development of more advanced and competent methods for solving fuzzy differential equations (Kaleva 1987; Friedman et al. 1999; Chalco-Cano and Roman-Flores 2006;  Tapaswini and Chakraverty 2013), fuzzy integral equations (Otadi and Mosleh 2014; Ghanbari 2010). Various details on calculus (Silverman 1985) and integral equations (Wazwaz 2011) are also found in literature. In this point of view, we present an appropriate numerical procedure for solving nonlinear fuzzy integro-differential equations. We consider the nonlinear Fredholm and Volterra integro-differential equations of the second kind. We use parametric forms of fuzzy numbers to convert nonlinear fuzzy integro-differential equations to a nonlinear system of integro-differential equations in crisp case.

The technique we use is the homotopy perturbation method (HPM). It is a general analytical procedure expansively applied for solving nonlinear equations as well as initial and boundary value problems which has been widely developed by scientists and engineers. HPM was developed by He (1999) and later promote the growth of it in various stages (He 2000, 2003, 2004) consistently. This technique is the coupling of the traditional perturbation method and homotopy in topology. It continuously deforms the intricate problem into a simple problem which is free from constraints and simple to solve without any need to transform nonlinear terms. Studies on integration using HPM is also made (Chun 2007). Applications of HPM among researchers has been tremendously increased over the last decades as it a powerful tool handler for solving functional integral equations (Abbasbandy 2007), singularly perturbed Volterra integral equations (Alnasr and Momani 2008), nonlinear integral and integro-differential equations (Saberi-Nadjafi and Ghorbani 2009), fuzzy integral equations (Matinfar and Saeidy 2010), Lotka–Volterra equations (Chowdhury and Rahman 2012). A review of the recently developed works using HPM can be found in (Demir et al. 2013; Filobello-Nino et al. 2014a, b; Vazquez-Leal and Sarmiento-Reyes 2015; Narayanamoorthy and Sathiyapriya 2016). Following this, a new approach for solving fractional PDEs arising mathematical physics by employing local fractional homotopy perturbation method is also proposed (Yang et al. 2015a). Moreover, authors are also referred to few recent papers where HPM serves as an existing background in finding solutions of fractional boundary value problems (Yang et al. 2015b) local fractional diffusion equation (Yang et al. 2016) and local fractional nonlinear PDEs (Zhang and Yang 2016). In this way, various works related to HPM is reported frequently as homotopy perturbation method is considered to be one of the most powerful methods to handle a wide variety of real problems arising in different fields. Hence HPM is of utmost interest to many researchers and scientists.

As it is extensively known that the importance of research on nonlinear integro-differential equations is that many observable facts, practical or theoretical is of nonlinear nature. Hence various other methods for solving them such as using fixed point theorems (Rahimi et al. 2011), expansion method (Allahviranloo et al. 2014), differential transform method (Behiry and Mohamed 2012), laplace transformation method (Das and Talukdar 2014) and homotopy analysis method (Hussain and Ali 2013) were also reported in recent times. We also referred an article (Atangana 2015) which presented a novel method for the lassa hemorrhagic fever. Using the fact that HPM is valid uniformly even for large parameters and is more accurate than the perturbation solutions as well as it eliminates the shortcomings arising in the small parameter assumption lead us to the development of our proposed method. Considering all the aforementioned factors, we intend to present a pertinent numerical approach for solving nonlinear fuzzy integro-differential equations of the second kind and find fuzzy approximate solutions to them.

The paper is organized as follows: In the section ‘Preliminaries’, some background materials needed for fuzzy operations are brought. Then the nonlinear fuzzy integro-differential equations are discussed with the requisite lemmas. ‘Analysis of HPM’ section presents the basic idea of the method. Our key findings are given in ‘Description of the proposed approximation technique’. It is followed by ‘Stability analysis’ and ‘Convergent Analysis’ section provided in detail for proving the competence of the proposed technique. Further ‘Numerical illustrations’ section are included and finally ‘Conclusion’ section is provided.

## Preliminaries

In this section, some basic notations and definitions that are used in fuzzy operations are summarized as follows.

### **Definition 1**

(Dubois and Prade 1982) A fuzzy number is a function $u{:}R \to I =$ [0, 1] satisfying the following properties:

1. $$u$$ is normal, i.e., $$\exists$$$$x_{0}$$$$\in R$$ with $$u\left( {x_{0} } \right) = 1$$.
2. $u$ is a convex fuzzy set (i.e., $u\left( {\lambda x + \left( {1 - \lambda } \right)y} \right) \ge min\left\{ {u\left( x \right), u\left( y \right)} \right\} \forall x,y \in R, \lambda \in \left[ {0, 1} \right]).$
3. $u$ is upper semi-continuous on $R$.
4. $\left\{ { \overline{x \in R:u\left( x \right) > 0} } \right\}$ is compact, where $\bar{A}$ denotes the closure of $A$.

The set of all fuzzy numbers is denoted by $E$. Obviously $R \subset E$ and it is understood as $R = \left\{ {\chi_{x} : \chi } \right.$ is usual real number}. For $0 \le \alpha \le 1$, denote $\left[ u \right]_{r} = \left\{ {x \in R;u\left( x \right) \ge r} \right\}$ and $\left[ u \right]_{0} = \left\{ { \overline{x \in R:u\left( x \right) > 0} } \right\}.$ Then it is well-known that for any $\alpha \in \left[ {0,1} \right]$, $\left[ u \right]_{r}$ is a bounded closed interval.

### **Definition 2**

(Goetschel and Voxman 1986) For arbitrary fuzzy numbers $u = \left( { {\underline{u}}, \bar{u}} \right)$ and $v = \left( {\underline{v} , \bar{v}} \right)$ the quantity

$$D\left( {u,v} \right) = max\left\{ { \mathop{sup}\limits_{\left( { 0 \le \alpha \le 1} \right)} \left| { \underline{u} \left( \alpha \right) - \underline{v} \left( \alpha \right)} \right|, \mathop{sup} \limits_{\left( { 0 \le \alpha \le 1} \right)} \overline{|u} \left( \alpha \right) - \bar{v}\left( \alpha \right)| } \right\},$$

is the Hausdorff distance between $u$ and $v$.

### **Definition 3**

(Seikkala 1987) Let $$I$$ be a real interval. A mapping $$\bar{v}:I \to E$$ is called a fuzzy process and we denote the $$\alpha$$-level set by $$\left[v\left( t \right)\right]_{\alpha } =$$$$\underline{v} \left( {t,\alpha } \right),\bar{v}\left( {t,\alpha } \right)]$$. The seikkala derivative $$\widetilde{{v^{\prime}}}\left( t \right)$$ of $$\tilde{v}$$ is defined by

$$\left[ {v'\left( t \right)}\right]_{\alpha } = \left[\underline{{v^{\prime}}} \left( {t,\alpha } \right),\bar{v}^{'} \left( {t,\alpha } \right)\right],$$

provided that is a equation defines a fuzzy number $\widetilde{{v^{\prime}}}\left( t \right) \in E.$

### **Definition 4**

(Seikkala 1987) The fuzzy integral of a fuzzy process $\tilde{v}$, $\int\nolimits_{a}^{b} v\left( t \right)dt$ for $a,b \in I$, is defined by

$$\left[ {\int\nolimits_{a}^{b} v\left( t \right)dt} \right]_{\alpha } = \left[ {\int\nolimits_{a}^{b} {\underline{v}} \left( {t,\alpha } \right)dt, \int\nolimits_{a}^{b} \bar{v}\left( {t,\alpha } \right)dt} \right],$$

provided that the Lebesgue integrals on the right exist.

### **Definition 5**

(Puri and Ralescu 1983) A function $f:\left( {a,b} \right) \to E^{1}$ is called H-differentiable at $\hat{x} \in \left( {a,b} \right)$ if, for $h > 0$ sufficiently small, there exist the H-differences $f\left( {\hat{x} + h} \right) - f\left( {\hat{x}} \right)$, $f\left( {\hat{x}} \right) - f\left( {\hat{x} - h} \right)$, and an element $f'\left( {\hat{x}} \right) \in E^{1}$ such that: $\mathop{lim} \nolimits_{h \to 0^{ + } } D\left( {\frac{{f\left( {\hat{x} + h} \right) - f\left( {\hat{x}} \right)}}{h}, f'\left( {\hat{x}} \right)} \right) = \mathop{lim} \nolimits_{h \to 0^{ - } } D\left( {\frac{{f\left( {\hat{x}} \right) - f\left( {\hat{x} - h} \right)}}{h}, f'\left( {\hat{x}} \right)} \right) = 0$.

Then $f'\left( {\hat{x}} \right)$ is called the fuzzy derivative of $f$ at $\left( {\hat{x}} \right)$.

### Nonlinear fuzzy integro-differential equations

In this section, we discuss the nonlinear Fredholm integro-differential equations of the second kind (Hochstadt 1973) and is given by

1 $$F'\left( x \right) = f\left( x \right) + \lambda \int\nolimits_{a}^{b} k\left( {x,t, F\left( t \right)} \right)F'\left( t \right)dt,\quad F\left( {x_{0} } \right) = X_{0}$$

where $\lambda > 0$, $a$ and $b$ are constants, $k\left( {x,t} \right)$ is an arbitrary continuous kernel function over the square $a \le x$, $t \le b$ and $f\left( x \right)$ is a function of $a \le x \le b$. If $F$ is a fuzzy function of $x \in \left[ {a,b} \right]$ and $F'$ is the fuzzy derivative (By definition 5), this equation may possess only fuzzy solution. Sufficient condition for the existence equation of the second kind can be found in (Balasubramaniam and Muralisankar 2001). Let $F\left( x \right) = \left( {{\underline{F}} \left( {x, \alpha } \right), \bar{F}\left( {x, \alpha } \right)} \right)$ is a fuzzy solution of Eq. (1) and hence we have the equivalent system of nonlinear fuzzy Fredholm integro-differential equations of the second kind (FFIDE-2) and is given as follows.

2 $${\underline{F}}^{'} \left( x \right) = \underline{f } \left( x \right) + \lambda \int\nolimits_{a}^{b} \underline{{k\left( {x,t, F\left( t \right)} \right)F'\left( t \right)}} dt,\,\,\,{\underline{F}} \left( {x_{0} } \right) = \underline{{X_{0} }}$$

3 $$\overline{{F^{\prime}}} \left( x \right) = \bar{f} \left( x \right) + \lambda \int\nolimits_{a}^{b} \overline{{k\left( {x,t, F\left( t \right)} \right)F'\left( t \right)}} dt,\,\,\,\bar{F} \left( {x_{0} } \right) = \overline{{X_{0} }}$$

which possesses a unique solution $\left( {\underline{F } ,\bar{F}} \right) \in B$ which is a fuzzy function, i.e., for each $x$, the pair $\left( {\underline{F } \left( {x,\alpha } \right),\bar{F}\left( {x,\alpha } \right)} \right)$ is a fuzzy number. The parametric form of the above equations are given by

4 $${\underline{F}}^{'} \left( {x,\alpha } \right) = \underline{f } \left( {x,\alpha } \right) + \lambda \int\nolimits_{a}^{b} \underline{{k\left( {x,t, F\left( {t,\alpha } \right)} \right)F'\left( {t,\alpha } \right)}} dt, {\underline{F}} \left( {x_{0} } \right) = {\underline{X}}_{0} \left( \alpha \right)_{ }$$

5 $$\overline{{F^{\prime}}} \left( {x,\alpha } \right) = \bar{f} \left( {x,\alpha } \right) + \lambda \int\nolimits_{a}^{b} \overline{{k\left( {x,t, F\left( {t,\alpha } \right)} \right)F'\left( {t,\alpha } \right)}} dt, \bar{F} \left( {x_{0} } \right) = \bar{X}_{0} \left( \alpha \right)$$

for $$\alpha \in \left[ {0,1} \right]$$, where

6 $$\underline{{k\left( {x,t, F\left( {t,\alpha } \right)} \right)F'\left( {t,\alpha } \right)}} = \left\{ {\begin{array}{ll} {k\left( {x,t} \right) {\underline{F}} \left( {t, \alpha } \right){\underline{F}}^{'} \left( {t, \alpha } \right) k\left( {x, t} \right) \ge 0} \\ {k\left( {x, t} \right)\bar{F}\left( {t, \alpha } \right)\overline{{F^{\prime}}} \left( {t, \alpha } \right) k\left( {x, t} \right) < 0} \\ \end{array} } \right.$$

and

7 $$\overline{{k\left( {x, t} \right) F\left( {t, \alpha } \right)F'\left( {t,\alpha } \right)}} = \left\{ {\begin{array}{ll} {k\left( {x,t} \right) \bar{F}\left( {t, \alpha } \right)\overline{{F^{\prime}}} \left( {t, \alpha } \right) k\left( {x, t} \right) \ge 0} \\ {k\left( {x, t} \right){\underline{F}} \left( {t, \alpha } \right){\underline{F}}^{'} \left( {t, \alpha } \right) k\left( {x, t} \right) < 0} \\ \end{array} } \right.$$

The nonlinear Volterra integro-differential equations of the second kind is given by

8 $$F'\left( x \right) = f\left( x \right) + \lambda \int\nolimits_{a}^{x} k\left( {x,t, F\left( t \right)} \right)F'\left( t \right)dt,\,\,F\left( {x_{0} } \right) = X_{0}$$

Here upper limit $x$ is a variable, where $x > t$, $x \in \left[ {a,b} \right]$ and $b > \infty$. The equivalent system of the nonlinear fuzzy Volterra integro-differential equations of the second kind (FVIDE-2) and its parametric forms are straightforward.

#### **Lemma**

(Ralescu 1979) *Let*$\left[ {{\underline{v}} \left( {t,\alpha } \right),\bar{v}\left( {t,\alpha } \right)} \right]$, $\alpha \in \left( {0,1} \right]$*, be a given family of non-empty intervals. If* (1) $\left[ { {\underline{v}} \left( \alpha \right),\bar{v}\left( \alpha \right)\left] \supset \right[{\underline{v}} \left( \beta \right),\bar{v}\left( \beta \right)} \right]$*for*$0 < \alpha \le \beta$*and* (2) $\left[ \mathop{lim} \nolimits_{k \to \infty } {\underline{v}} \left( \alpha_{k} \right), \mathop{lim} \nolimits_{k \to \infty } \,\bar{v}\left( \alpha_{k} \right) \right] = \left[ { {\underline{v}} \left( \alpha \right),\bar{v}\left( \alpha \right)} \right]$*whenever*$(\alpha_{k} )$*is a non-decreasing sequence converging to*$\alpha \in \left( {0,1} \right]$*, then the family*$\left[ { {\underline{v}} \left( \alpha \right),\bar{v}\left( \alpha \right)} \right]$, $0 < \alpha \le 1$*, are the*$\alpha -$*level sets of a fuzzy number*$v$ in $E$*. Conversely if*$\left[ { {\underline{v}} \left( \alpha \right),\bar{v}\left( \alpha \right)} \right]$, $0 < \alpha \le 1$*, are the*$\alpha -$*level sets of a fuzzy number*$\tilde{v}$ in $E$, *then the conditions (1) and (2) holds true.*

#### **Lemma**

(Bede and Gal 2005) *For*$\widetilde{{x_{0} }} \in R$*the fuzzy differential equation*

$$\tilde{y}' = \tilde{f}\left( {x,y} \right),\,\,\,\,\tilde{y}\left( {x_{0} } \right) = \tilde{y} \in E$$

*where*$\tilde{f}:R \times E \to E$*is supposed to be continuous, if equivalent to one of the integral equations:*

*(1)*$$\tilde{y}\left( x \right) = \widetilde{{y_{0} }} + \int\nolimits_{{x_{0} }}^{x} f(t, \tilde{y}\left( {t)} \right)$$, $$\forall x \in \left[ {x_{0} , x_{1} } \right]$$

*Or (2)*$\tilde{y}\left( x \right) = \widetilde{{y_{0} }} + \left( { - 1} \right)\int\nolimits_{{x_{0} }}^{x} f(t, \tilde{y}\left( {t)} \right)$, $\forall x \in \left[ {x_{0} , x_{1} } \right]$*on some interval*$\left( {x_{0} , x_{1} } \right)$*under the differentiability condition, (1) or (2) respectively*.

### Analysis of homotopy perturbation method

The essential idea of this method is to introduce a homotopy parameter, say $p$, which takes the values from 0 to 1. When $p = 0$, the system of equation usually reduces to a sufficiently simplified form, which normally admits a rather simple solution. As $p$ gradually increases to 1, the system goes through a sequence of deformation, the solution of each of which is close to that at the previous stage of deformation. Eventually at $p = 1$, the system takes the original form of the equation and final stage of deformation gives the desired solution. To illustrate HPM, consider the nonlinear differential equation (Demir et al. 2013)

9 $$A\left( u \right) - f\left( r \right) = 0,\,\,\,r \in \varOmega ,$$

with boundary conditions $B\left( {u, \frac{\partial u}{\partial n}} \right) = 0,\,\,\,r \in \varGamma ,$ where $A\left( u \right) = L\left( u \right) + N\left( u \right)$, $L$ is a linear operator, $N$ is a nonlinear operator, $B$ is a boundary operator, $\varGamma$ is the boundary of the domain $\varvec{\varOmega}$ and $f\left( r \right)$ is a known analytic function. In order to use the HPM, a suitable construction of homotopy is of vital importance. He (1999, 2000) constructed a homotopy $U:\varOmega \times \left[ {0,1} \right]$ that satisfies

10 $$H\left( {U,p} \right) = \left( {1 - p} \right)\left[ {L\left( U \right) - L\left( {u_{0} } \right)} \right] + p\left[ {A\left( U \right) - f\left( r \right)} \right] = 0$$

or

11 $$H\left( {U,p} \right) = L\left( U \right) - L\left( {u_{0} } \right) + p\left[ {L\left( {u_{0} } \right) + N\left( U \right) - f\left( r \right)} \right] = 0$$

where $r \in \varOmega$ and $p \in \left[ {0, 1} \right]$ is called homotopy parameter and $u_{0}$ is an initial approximation of Eq. (9). It is obvious that

12 $$H\left( {U,0} \right) = L\left( U \right) - L\left( {u_{0} } \right) = 0,\,\,\,H\left( {U,1} \right) = A\left( u \right) - f\left( r \right) = 0,$$

and the changing process of $p$ from 0 to 1, is just that of $H\left( {U,p} \right)$ from $L\left( U \right) - L\left( {u_{0} } \right)$ to $A\left( U \right) - f\left( r \right)$ and this deformation is called homotopy in topology. Applying HPM, the solution of Eqs. (10) or (11) can be expressed as a series in $p$, where $0 \le p \le 1$, is

13 $$u = u_{0} + p^{1} u_{1} + p^{2} u_{2} + \cdots$$

When $p \to 1$, Eq. (10) or Eq. (11) corresponds to Eq. (9) and becomes the approximate solution of Eq. (9), i.e.,

14 $$U = \mathop{lim} \limits_{p \to 1} u = \sum\limits_{n = 0}^{\infty } u_{n} = u_{0} + u_{1} + u_{2} + \cdots$$

The above series is convergent for most of the cases and the rate of convergence depends on $L\left( u \right)$ (He 2000).

### Description of the proposed approximation technique

One of the main aims of this paper is to introduce an approximation technique by extending the analysis of homotopy perturbation method, for solving nonlinear fuzzy Fredholm integro-differential equations of the second kind where its general form is given as

15 $$F'\left( x \right) = f\left( x \right) + \lambda \int\nolimits_{a}^{b} k\left( {x,t, F\left( t \right)} \right)F'\left( t \right)dt,\quad F\left( {x_{0} } \right) = X_{0}$$

$$\lambda > 0$$, $$a \le x \le b$$, $$0 \le \alpha \le 1$$ where $$F'\left( x \right) = \left( {{\underline{F}}^{'} \left( {x, \alpha } \right), \overline{{F^{\prime}}} \left( {x, \alpha } \right)} \right)$$, $$f\left( x \right) = \left( {{\underline{f}} \left( {x, \alpha } \right), \bar{f}\left( {x, \alpha } \right)} \right)$$, $$k\left( {x,t} \right) = \left( {{\underline{k}} \left( {x, t} \right), \bar{k}\left( {x, t} \right)} \right)$$, $$F\left( t \right) = \left( {{\underline{F}} \left( {t, \alpha } \right), \bar{F}\left( {t, \alpha } \right)} \right)$$ and $$F'\left( t \right) = \left( {{\underline{F}}^{'} \left( {t, \alpha } \right), \overline{{F^{\prime}}} \left( {t, \alpha } \right)} \right)$$.

We consider the above initial value problem with the arbitrary continuous kernels of the form $k\left( {x,t} \right) = g\left( x \right)h\left( t \right) = \sum\nolimits_{i = 0}^{\infty } g_{i} \left( x \right)h_{i} \left( t \right)$. By using HPM, we can have

16 $$({\underline{F}} \left( u \right), \bar{F}\left( u \right) = \left\{ {\begin{array}{*{20}c} {{\underline{u}} \left( {x, \alpha } \right)} \\ {\bar{u}\left( {x, \alpha } \right)} \\ \end{array} } \right.$$

17 $$({\underline{L}} \left( u \right), \bar{L}\left( u \right) = \left\{ \begin{array}{l} {{\underline{F}}}^{'} \left( {x,\alpha } \right) - {\underline{f}} \left( {x, \alpha } \right) = 0 \\ \overline{F}^{'} \left( {x,\alpha } \right) - \bar{f}\left( {x, \alpha } \right) = 0 \\ \end{array} \right.$$

Hence we can define the convex homotopy as follows

18 $$H\left( {u,p,\alpha } \right) = \left\{ {\begin{array}{*{20}c} {{\underline{u}}^{'} \left( {x,\alpha } \right) - {\underline{f}} \left( {x, \alpha } \right) - p\int\nolimits_{a}^{b} {\underline{g}} \left( x \right){\underline{h}} \left( t \right){\underline{u}} \left( {t,\alpha } \right){\underline{u}} '\left( {t,\alpha } \right)dt} \\ {\bar{u}^{'} \left( {x,\alpha } \right) - \bar{f}\left( {x, \alpha } \right) - p\int\nolimits_{a}^{b} \bar{g}\left( x \right)\bar{h}\left( t \right)\bar{u}\left( {t,\alpha } \right)\bar{u}^{'} \left( {t,\alpha } \right)dt} \\ \end{array} } \right.$$

and continuously trace an implicitly defined curve from a starting point $H\left( {u,0,\alpha } \right)$ to a solution function $H\left( {u,1,\alpha } \right)$, where $0 \le \alpha \le 1$. Solution of the above equations can be readily assumed as

19 $$\left\{ {\begin{array}{*{20}c} {{\underline{u}} \left( {x, \alpha } \right) = \sum\nolimits_{i = 0}^{\infty } p^{i} {\underline{u}}_{i} \left( {x, \alpha } \right)} \\ {\bar{u}\left( {x, \alpha } \right) = \sum\nolimits_{i = 0}^{\infty } p^{i} \bar{u}_{i} \left( {x, \alpha } \right)} \\ \end{array} } \right.$$

where (${\underline{u}}_{i} , \bar{u}_{i} ) \forall i$ are unknown functions to be determined.

The initial approximation can be taken as

20 $$p^{0} :\left\{ {\begin{array}{*{20}c} {{\underline{u}}^{\varvec{'}}_{0} \left( {x,\alpha } \right) - {\underline{f}} \left( {x,\alpha } \right) = 0 \Rightarrow {\underline{u}}^{\varvec{'}}_{0} \left( {x,\alpha } \right) = {\underline{f}} \left( {x,\alpha } \right)} \\ {\overline{{u^{\prime}}}_{0} \left( {x,\alpha } \right) - \bar{f}\left( {x,\alpha } \right) = 0 \Rightarrow \overline{{u^{\prime}}}_{0} \left( {x,\alpha } \right) = \bar{f}\left( {x,\alpha } \right)} \\ \end{array} } \right.$$

21 $$\left\{ {\begin{array}{*{20}c} {{\underline{u}}_{0} \left( {x,\alpha } \right) = \int\nolimits_{a}^{b} {\underline{f}} \left( {x,\alpha } \right)dx} \\ {\bar{u}_{0} \left( {x,\alpha } \right) = \int\nolimits_{a}^{b} \bar{f}\left( {x,\alpha } \right)dx} \\ \end{array} } \right.$$

Substitute Eq. (20) into Eq. (18) and equating the coefficients with identical powers of $p$, we have

22 $$p^{1} :\left\{ {\begin{array}{*{20}c} {{\underline{u}}^{\varvec{'}}_{1} \left( {x,\alpha } \right) = \int\nolimits_{a}^{b} {\underline{g}} \left( x \right){\underline{h}} \left( t \right){\underline{u}}_{0} \left( {t,\alpha } \right){\underline{u}}^{\varvec{'}}_{0} \left( {t,\alpha } \right)dt} \\ {\overline{{u^{\prime}}}_{1} \left( {x,\alpha } \right) = \int\nolimits_{a}^{b} \bar{g}\left( x \right)\bar{h}\left( t \right)\bar{u}_{0} \left( {t,\alpha } \right)\overline{{u^{\prime}}}_{0} \left( {t,\alpha } \right)dt} \\ \end{array} } \right.$$

23 $$\left\{ {\begin{array}{*{20}c} {{\underline{u}}_{1} \left( {x,\alpha } \right) = \int\nolimits_{a}^{b} (\int\nolimits_{a}^{b} {\underline{g}} \left( x \right){\underline{h}} \left( t \right){\underline{u}}_{0} \left( {t,\alpha } \right){\underline{u}}^{\varvec{'}}_{0} \left( {t,\alpha } \right)dt)dx} \\ {\bar{u}_{1} \left( {x,\alpha } \right) = \int\nolimits_{a}^{b} (\int\nolimits_{a}^{b} \bar{g}\left( x \right)\bar{h}\left( t \right)\bar{u}_{0} \left( {t,\alpha } \right)\overline{{u^{\prime}}}_{0} \left( {t,\alpha } \right)dt)dx} \\ \end{array} } \right.$$

24 $$p^{2} :\left\{ {\begin{array}{*{20}c} {{\underline{u}}^{\varvec{'}}_{2} \left( {x,\alpha } \right) = \int\nolimits_{a}^{b} {\underline{g}} \left( x \right){\underline{h}} \left( t \right)\left( {{\underline{u}}_{0} \left( {t,\alpha } \right){\underline{u}}^{\varvec{'}}_{1} \left( {t,\alpha } \right) + {\underline{u}}_{1} \left( {t,\alpha } \right){\underline{u}}^{\varvec{'}}_{0} \left( {t,\alpha } \right)} \right)dt} \\ {\overline{{u^{\prime}}}_{2} \left( {x,\alpha } \right) = \int\nolimits_{a}^{b} \bar{g}\left( x \right)\bar{h}\left( t \right)\left( {\bar{u}_{0} \left( {t,\alpha } \right)\overline{{u^{\prime}}}_{1} \left( {t,\alpha } \right) + \bar{u}_{1} \left( {t,\alpha } \right)\overline{{u^{\prime}}}_{0} \left( {t,\alpha } \right)} \right)dt} \\ \end{array} } \right.$$

25 $$\left\{ {\begin{array}{*{20}c} {{\underline{u}}_{2} \left( {x,\alpha } \right) = \int\nolimits_{a}^{b} (\int\nolimits_{a}^{b} {\underline{g}} \left( x \right){\underline{h}} \left( t \right)\left( {{\underline{u}}_{0} \left( {t,\alpha } \right){\underline{u}}^{\varvec{'}}_{1} \left( {t,\alpha } \right) + {\underline{u}}_{1} \left( {t,\alpha } \right){\underline{u}}^{\varvec{'}}_{0} \left( {t,\alpha } \right)} \right)dt)dx} \\ {\bar{u}_{2} \left( {x,\alpha } \right) = \int\nolimits_{a}^{b} (\int\nolimits_{a}^{b} \bar{g}\left( x \right)\bar{h}\left( t \right)\left( {\bar{u}_{0} \left( {t,\alpha } \right)\overline{{u^{\prime}}}_{1} \left( {t,\alpha } \right) + \bar{u}_{1} \left( {t,\alpha } \right)\overline{{u^{\prime}}}_{0} \left( {t,\alpha } \right)} \right)dt)dx} \\ \end{array} } \right.$$

26 $$p^{3} :\left\{ {\begin{array}{*{20}c} {{\underline{u}}^{\varvec{'}}_{3} \left( {x,\alpha } \right) = \int\nolimits_{a}^{b} {\underline{g}} \left( x \right){\underline{h}} \left( t \right)\left( {{\underline{u}}_{0} \left( {t,\alpha } \right){\underline{u}}^{\varvec{'}}_{2} \left( {t,\alpha } \right) + {\underline{u}}_{1} \left( {t,\alpha } \right){\underline{u}}^{\varvec{'}}_{1} \left( {t,\alpha } \right) + {\underline{u}}^{\varvec{'}}_{2} \left( {t,\alpha } \right){\underline{u}}^{\varvec{'}}_{0} \left( {t,\alpha } \right)} \right)dt} \\ {\overline{{u^{\prime}}}_{3} \left( {x,\alpha } \right) = \int\nolimits_{a}^{b} \bar{g}\left( x \right)\bar{h}\left( t \right)\left( {\bar{u}_{0} \left( {t,\alpha } \right)\overline{{u^{\prime}}}_{2} \left( {t,\alpha } \right) + \bar{u}_{1} \left( {t,\alpha } \right)\overline{{u^{\prime}}}_{1} \left( {t,\alpha } \right) + \bar{u}^{\varvec{'}}_{2} \left( {t,\alpha } \right)\bar{u}^{\varvec{'}}_{0} \left( {t,\alpha } \right)} \right)dt} \\ \end{array} } \right.$$

27 $$\left\{ {\begin{array}{*{20}c} {{\underline{u}}_{3} \left( {x,\alpha } \right) = \int\nolimits_{a}^{b} (\int\nolimits_{a}^{b} {\underline{g}} \left( x \right){\underline{h}} \left( t \right)\left( {{\underline{u}}_{0} \left( {t,\alpha } \right){\underline{u}}^{\varvec{'}}_{2} \left( {t,\alpha } \right) + {\underline{u}}_{1} \left( {t,\alpha } \right){\underline{u}}^{\varvec{'}}_{1} \left( {t,\alpha } \right) + {\underline{u}}_{2} \left( {t,\alpha } \right){\underline{u}}^{\varvec{'}}_{0} \left( {t,\alpha } \right)} \right)dt)dx} \\ {\bar{u}_{3} \left( {x,\alpha } \right) = \int\nolimits_{a}^{b} (\int\nolimits_{a}^{b} \bar{g}\left( x \right)\bar{h}\left( t \right)\left( {\bar{u}_{0} \left( {t,\alpha } \right)\overline{{u^{\prime}}}_{2} \left( {t,\alpha } \right) + \bar{u}_{1} \left( {t,\alpha } \right)\overline{{u^{\prime}}}_{1} \left( {t,\alpha } \right) + + \bar{u}_{2} \left( {t,\alpha } \right)\bar{u}^{\varvec{'}}_{0} \left( {t,\alpha } \right)} \right)dt)dx} \\ \end{array} } \right.$$

In the same way, we get $p^{4}$ as follows.

28 $$\left\{ {\begin{array}{*{20}c} {{\underline{u}}_{4}^{\varvec{'}} \left( {x,\alpha } \right) = \int\nolimits_{a}^{b} {\underline{g}} \left( x \right){\underline{h}} \left( t \right)\left( {{\underline{u}}_{0} \left( {t,\alpha } \right){\underline{u}}^{\varvec{'}}_{3} \left( {t,\alpha } \right) + {\underline{u}}_{1} \left( {t,\alpha } \right){\underline{u}}^{\varvec{'}}_{2} \left( {t,\alpha } \right) + {\underline{u}}_{2} \left( {t,\alpha } \right){\underline{u}}^{\varvec{'}}_{1} \left( {t,\alpha } \right) + u_{3} \left( {t,\alpha } \right){\underline{u}}_{0}^{\varvec{'}} \left( {t,\alpha } \right)} \right)dt)dx} \\ {\bar{u}_{4}^{\varvec{'}} \left( {x,\alpha } \right) = \int\nolimits_{a}^{b} \bar{g}\left( x \right)\bar{h}\left( t \right)\left( {\bar{u}_{0} \left( {t,\alpha } \right)\overline{u}_{3}^{\varvec{'}} \left( {t,\alpha } \right) + \bar{u}_{1} \left( {t,\alpha } \right)\overline{u}_{3}^{\varvec{'}} \left( {t,\alpha } \right) + + \bar{u}_{2} \left( {t,\alpha } \right)\bar{u}^{\varvec{'}}_{1} \left( {t,\alpha } \right) + \bar{u}_{3} \left( {t,\alpha } \right)\bar{u}^{\varvec{'}}_{0} \left( {t,\alpha } \right)} \right)dt)dx} \\ \end{array} } \right.$$

29 $$\left\{ {\begin{array}{*{20}c} {{\underline{u}}_{4} \left( {x,\alpha } \right) = \int\nolimits_{a}^{b} (\int\nolimits_{a}^{b} {\underline{g}} \left( x \right){\underline{h}} \left( t \right)\left( {{\underline{u}}_{0} \left( {t,\alpha } \right){\underline{u}}^{\varvec{'}}_{3} \left( {t,\alpha } \right) + {\underline{u}}_{1} \left( {t,\alpha } \right){\underline{u}}^{\varvec{'}}_{2} \left( {t,\alpha } \right) + {\underline{u}}_{2} \left( {t,\alpha } \right){\underline{u}}^{\varvec{'}}_{1} \left( {t,\alpha } \right) + {\underline{u}}_{3} \left( {t,\alpha } \right){\underline{u}}^{\varvec{'}}_{0} \left( {t,\alpha } \right)} \right)dt)dx} \\ {\bar{u}_{4} \left( {x,\alpha } \right) = \int\nolimits_{a}^{b} (\int\nolimits_{a}^{b} \bar{g}\left( x \right)\bar{h}\left( t \right)\left( {\bar{u}_{0} \left( {t,\alpha } \right)\overline{u}_{3}^{\varvec{'}} \left( {t,\alpha } \right) + \bar{u}_{1} \left( {t,\alpha } \right)\overline{u}_{2}^{\varvec{'}} \left( {t,\alpha } \right) + + \bar{u}_{2} \left( {t,\alpha } \right)\bar{u}^{\varvec{'}}_{1} \left( {t,\alpha } \right) + \bar{u}_{3} \left( {t,\alpha } \right)\bar{u}^{\varvec{'}}_{0} \left( {t,\alpha } \right)} \right)dt)dx} \\ \end{array} } \right.$$

and so on. Therefore the solution of Eq. (15) can be obtained as

30 $$\left\{ {\begin{array}{*{20}c} {{\underline{u}} \left( {x, \alpha } \right) = \sum\nolimits_{i = 0}^{\infty } {\underline{u}}_{i} \left( {x, \alpha } \right)} \\ {\bar{u}\left( {x, \alpha } \right) = \sum\nolimits_{i = 0}^{\infty } \bar{u}_{i} \left( {x, \alpha } \right)} \\ \end{array} } \right.$$

Since the above series is infinite, all the terms of the series cannot be determined in practice and so we use an appropriate approximation of the solution by the following truncated series

31 $$\left( {{\underline{\gamma }}_{m} \left( x \right), \bar{\gamma }_{m} \left( x \right)} \right) = \left( {\sum\limits_{i = 0}^{m - 1} {\underline{u}}_{i} \left( {x, \alpha } \right),\sum\limits_{i = 0}^{m - 1} \bar{u}_{i} \left( {x, \alpha } \right)} \right)$$

with

32 $$\left( {{\underline{u}} \left( x \right), \bar{u}\left( x \right)} \right) = \left( {\mathop{lim} \limits_{m \to \infty } {\underline{\gamma }}_{m} \left( {x, \alpha } \right),\mathop{lim} \limits_{m \to \infty } \bar{\gamma }_{m} \left( {x, \alpha } \right)} \right).$$

The similar algorithm can be applied for nonlinear fuzzy Volterra integro-differential equations of the second kind.

### Stability analysis

We present in this section, the general stability idea of the proposed numerical scheme for solving nonlinear fuzzy Fredholm integro-differential equation of the second kind. We consider the stability of the solution components $\left( {{\underline{u}} \left( {x, \alpha } \right),\bar{u}\left( {x, \alpha } \right)} \right)$ as given in Eq. (30) under the presence of a small perturbation in the function $\left( {{\underline{f}} \left( {x, \alpha } \right),\bar{f}\left( {x, \alpha } \right)} \right)$ which is used for initial fuzzy approximation as given in Eq. (21) is disturbed with the perturbation function $\left( {\underline{\delta f} \left( {x, \alpha } \right),\overline{\delta f} \left( {x, \alpha } \right)} \right)$ where it is an unknown function relative to $\left( {{\underline{f}} \left( {x, \alpha } \right),\bar{f}\left( {x, \alpha } \right)} \right)$. The following results can also be proved in a similar way for nonlinear FVIDE-2.

#### **Theorem 1**

*The presence of the small perturbation function*$\left( {\underline{\delta f} \left( {x, \alpha } \right),\overline{\delta f} \left( {x, \alpha } \right)} \right)$*in the continuous fuzzy function*$\left( {{\underline{f}} \left( {x, \alpha } \right),\bar{f}\left( {x, \alpha } \right)} \right)$*alters the fuzzy approximate solution *$\left( {{\underline{F}} \left( {x, \alpha } \right),\bar{F}\left( {x, \alpha } \right)} \right)$*by an equivalent value to the solution of nonlinear FFIDE-2* *Eq.* (15) *with initial fuzzy approximation equal to the perturbation function*$\left( {\underline{\delta f} \left( {x, \alpha } \right),\overline{\delta f} \left( {x, \alpha } \right)} \right)$*itself respectively.*

#### *Proof*

Without loss of generality, let us assume $\left( {{\underline{F}} \left( {x, \alpha } \right),\bar{F}\left( {x, \alpha } \right)} \right) = \left( {{\underline{u}} \left( {x, \alpha } \right),\bar{u}\left( {x, \alpha } \right)} \right)$ as the solution of Eq. (15) under the presence of a small perturbation in form of finite sequences given as follows

33 $$\left\{ {\begin{array}{*{20}c} {\underline{\delta f} \left( {x, \alpha } \right) = \left( { \underline{\delta f}_{1} \left( {x, \alpha } \right), \underline{\delta f}_{2} \left( {x, \alpha } \right), \ldots ,\underline{\delta f}_{n} \left( {x, \alpha } \right)} \right)} \\ {\overline{\delta f} \left( {x, \alpha } \right) = \left( {\overline{\delta f}_{1} \left( {x, \alpha } \right), \overline{\delta f}_{2} \left( {x, \alpha } \right), \ldots ,\overline{\delta f}_{n} \left( {x, \alpha } \right)} \right)} \\ \end{array} } \right.$$

then we have

34 $$\left\{ {\begin{array}{*{20}c} { {\underline{F}}^{\varvec{'}} \left( {x,\alpha } \right) = \underline{\delta f } \left( {x,\alpha } \right) + \int\nolimits_{a}^{b} \underline{{k\left( {x,t, F\left( {t,\alpha } \right)} \right)F'\left( {t,\alpha } \right)}} dt, {\underline{F}} \left( {x_{0} } \right) = {\underline{X }}_{0} \left( \alpha \right)_{ } } \\ {\overline{{F^{\prime}}} \left( {x,\alpha } \right) = \overline{\delta f} \left( {x,\alpha } \right) + \int\nolimits_{a}^{b} \overline{{k\left( {x,t, F\left( {t,\alpha } \right)} \right)F'\left( {t,\alpha } \right)}} dt, \bar{F} \left( {x_{0} } \right) = \bar{X}_{0} \left( \alpha \right)} \\ \end{array} } \right.$$

Now assume the initial fuzzy approximation as

35 $$\left\{ {\begin{array}{*{20}c} {\widetilde{{{\underline{V }} }}_{0} \left( {x,\alpha } \right) = \int\nolimits_{a}^{b} \left( {{\underline{F}}_{0} \left( {x,\alpha } \right) + {\underline{\varepsilon}}_{0} \left( {x,\alpha } \right) } \right)dx} \\ {\widetilde{{\bar{V}}}_{0} \left( {x,\alpha } \right) = \int\nolimits_{a}^{b} (\bar{F}_{0} \left( {x,\alpha } \right) + \bar{\varepsilon }_{0} \left( {x,\alpha } \right) )dx} \\ \end{array} } \right.$$

where

36 $$\left\{ {\begin{array}{*{20}c} {\widetilde{{{\underline{V }} }}_{0} \left( {x,\alpha } \right) = \left( { \widetilde{{{\underline{V }} }}_{10} \left( {x,\alpha } \right), \widetilde{{{\underline{V }} }}_{20} \left( {x,\alpha } \right), \ldots ,\widetilde{{{\underline{V }} }}_{n0} \left( {x,\alpha } \right)} \right)} \\ {\widetilde{{\bar{V}}}_{0} \left( {x,\alpha } \right) = \left( {\widetilde{{\bar{V}}}_{10} \left( {x,\alpha } \right), \widetilde{{\bar{V}}}_{20} \left( {x,\alpha } \right), \ldots ,\widetilde{{\bar{V}}}_{n0} \left( {x,\alpha } \right)} \right)} \\ \end{array} } \right.$$

37 $$\left\{ {\begin{array}{*{20}c} {\int\nolimits_{a}^{b} {\underline{F}}^{\varvec{'}}_{0} \left( {x,\alpha } \right)dx = \left( {\int\nolimits_{a}^{b} {\underline{F}}^{\varvec{'}}_{10} \left( {x,\alpha } \right)dx, \int\nolimits_{a}^{b} {\underline{F}}^{\varvec{'}}_{20} \left( {x,\alpha } \right)dx, \ldots ,\int\nolimits_{a}^{b} {\underline{F}}^{\varvec{'}}_{n0} \left( {x,\alpha } \right)dx} \right)} \\ {\int\nolimits_{a}^{b} \overline{F'}_{0} \left( {x,\alpha } \right)dx = \left( {\int\nolimits_{a}^{b} \overline{F'}_{10} \left( {x,\alpha } \right)dx,\int\nolimits_{a}^{b} \overline{F'}_{20} \left( {x,\alpha } \right)dx, \ldots ,\int\nolimits_{a}^{b} \overline{F'}_{n0} \left( {x,\alpha } \right)dx} \right)} \\ \end{array} } \right.$$

38 $$\left\{ {\begin{array}{*{20}c} {\int\nolimits_{a}^{b} {\underline{\varepsilon}}_{0} \left( {x,\alpha } \right)dx = \left( {\int\nolimits_{a}^{b} {\underline{\varepsilon}}_{10} \left( {x,\alpha } \right)dx, \int\nolimits_{a}^{b} {\underline{\varepsilon}}_{20} \left( {x,\alpha } \right)dx, \ldots ,\int\nolimits_{a}^{b} {\underline{\varepsilon}}_{n0} \left( {x,\alpha } \right)dx} \right)} \\ {\int\nolimits_{a}^{b} \bar{\varepsilon }_{0} \left( {x,\alpha } \right)dx = \left( {\int\nolimits_{a}^{b} \bar{\varepsilon }_{10} \left( {x,\alpha } \right)dx,\int\nolimits_{a}^{b} \bar{\varepsilon }_{20} \left( {x,\alpha } \right)dx, \ldots ,\int\nolimits_{a}^{b} \bar{\varepsilon }_{n0} \left( {x,\alpha } \right)dx} \right)} \\ \end{array} } \right.$$

We have by Eq. (22)

39 $$\left\{ {\begin{array}{*{20}c} {\widetilde{{{\underline{V }} }}_{1} \left( {x,\alpha } \right) = \int\nolimits_{a}^{b} \underline{{k\left( {x,t, (F\left( {t,\alpha } \right) + \xi_{0} } \right)\left( {F^{\prime}\left( {t,\alpha } \right) + \xi_{0} } \right)}} dt = {\underline{V }}_{1} \left( {x,\alpha } \right) + \int\nolimits_{a}^{b} {\underline{\varepsilon}}_{1} \left( {x,\alpha } \right)dx} \\ {\widetilde{{\bar{V}}}_{1} \left( {x,\alpha } \right) = \int\nolimits_{a}^{b} \overline{{k\left( {x,t, (F\left( {t,\alpha } \right) + \xi_{0} } \right)\left( {F^{\prime}\left( {t,\alpha } \right) + \xi_{0} } \right)}} dt = \bar{V}_{1} \left( {x,\alpha } \right) + \int\nolimits_{a}^{b} \bar{\varepsilon }_{1} \left( {x,\alpha } \right)dx} \\ \end{array} } \right.$$

Proceed by induction we have

40 $$\left\{ {\begin{array}{*{20}c} {\widetilde{{{\underline{V }} }}_{2} \left( {x,\alpha } \right) = {\underline{V }}_{2} \left( {x,\alpha } \right) + \int\nolimits_{a}^{b} {\underline{\varepsilon}}_{2} \left( {x,\alpha } \right)dx,} \\ {\widetilde{{\bar{V}}}_{2} \left( {x,\alpha } \right) = \bar{V}_{2} \left( {x,\alpha } \right) + \int\nolimits_{a}^{b} \bar{\varepsilon }_{2} \left( {x,\alpha } \right)dx} \\ \end{array} } \right.$$

41 $$\left\{ {\begin{array}{*{20}c} {\widetilde{{{\underline{V }} }}_{n} \left( {x,\alpha } \right) = {\underline{V }}_{n} \left( {x,\alpha } \right) + \int\nolimits_{a}^{b} {\underline{\varepsilon}}_{n} \left( {x,\alpha } \right)dx} \\ {\widetilde{{\bar{V}}}_{n} \left( {x,\alpha } \right) = \bar{V}_{n} \left( {x,\alpha } \right) + \int\nolimits_{a}^{b} \bar{\varepsilon }_{n} \left( {x,\alpha } \right)dx} \\ \end{array} } \right.$$

Hence the perturbed fuzzy approximate solution is given by

42 $$\left\{ {\begin{array}{*{20}c} {{\underline{F}} \left( {x, \alpha } \right) = \int\nolimits_{a}^{b} {\underline{F}}^{\varvec{'}} \left( {x,\alpha } \right)dx = \mathop{lim} \nolimits_{n \to \infty }\sum\nolimits_{i = 0}^{n} {\underline{V }}_{i} \left( {x,\alpha } \right)} \\ {\bar{F}\left( {x, \alpha } \right) = \int\nolimits_{a}^{b} \overline{{F^{\prime}}} \left( {x,\alpha } \right)dx = \mathop{lim} \nolimits_{n \to \infty }\sum\nolimits_{i = 0}^{n} \bar{V}_{i} \left( {x,\alpha } \right)} \\ \end{array} } \right.$$

Therefore the inclusion of the small perturbation function term $\left( {\int\nolimits_{a}^{b} {\underline{\varepsilon}}_{0} \left( {x,\alpha } \right)dx,\int\nolimits_{a}^{b} \bar{\varepsilon }_{0} \left( {x,\alpha } \right)dx} \right)$ affects the solution by

$$\left( {\int\nolimits_{a}^{b} {\underline{\varepsilon}}_{0} \left( {x,\alpha } \right)dx,\int\nolimits_{a}^{b} \bar{\varepsilon }_{0} \left( {x,\alpha } \right)dx} \right)$$

43 $$= \left( {\underline{{\tilde{f}}} \left( {x,\alpha } \right),\widetilde{{\bar{f}}}\left( {x,\alpha } \right)} \right) - \left( {{\underline{f}} \left( {x, \alpha } \right),\bar{f}\left( {x, \alpha } \right)} \right)$$

44 $$= \mathop{lim} \limits_{n \to \infty } \sum\limits_{i = 0}^{n} \left( {\widetilde{{{\underline{V }} }}_{j} \left( {x,\alpha } \right),\widetilde{{\bar{V}}}_{j} \left( {x,\alpha } \right)} \right) - \mathop{lim} \limits_{n \to \infty } \sum\limits_{i = 0}^{n} \left( {{\underline{V }}_{j} \left( {x,\alpha } \right),\bar{V}_{j} \left( {x,\alpha } \right)} \right)$$

45 $$= \mathop{lim} \limits_{n \to \infty }\sum\limits_{i = 0}^{n} \left( {\int\nolimits_{a}^{b} {\underline{\varepsilon}}_{j} \left( {x,\alpha } \right)dx,\int\nolimits_{a}^{b} \bar{\varepsilon }_{j} \left( {x,\alpha } \right)dx} \right)$$

From the above equation, we conclude that $\left( {\underline{\delta F} \left( {x, \alpha } \right),\overline{\delta F} \left( {x, \alpha } \right)} \right)$ and $\left( {\underline{\delta f} \left( {x, \alpha } \right),\overline{\delta f} \left( {x, \alpha } \right)} \right)$ are related by the generalized nonlinear FFIDE-2 as follows

46 $$\left\{ {\begin{array}{*{20}c} { \beta \underline{\delta F}^{\varvec{'}} \left( {x,\alpha } \right) = \underline{\delta f } \left( {x,\alpha } \right) + \int\nolimits_{a}^{b} \underline{{k\left( {x,t, \delta F\left( {t,\alpha } \right)} \right)\delta F'\left( {t,\alpha } \right)}} dt } \\ {\beta \overline{{\delta F^{\varvec{'}} }} \left( {x,\alpha } \right) = \overline{\delta f} \left( {x,\alpha } \right) + \int\nolimits_{a}^{b} \overline{{k\left( {x,t, \delta F\left( {t,\alpha } \right)} \right)\delta F'\left( {t,\alpha } \right)}} dt } \\ \end{array} } \right.$$

where $\beta$ is a constant.

As $\left( {\underline{\delta f} \left( {x, \alpha } \right),\overline{\delta f} \left( {x, \alpha } \right)} \right)$ is an unknown function and by taking supremum for it $\left( {\mathop{sup}\nolimits_{a \le x \le b}\left| {\underline{\delta f} \left( {x, \alpha } \right)} \right| < {\underline{\varepsilon}} , \mathop{sup}\nolimits_{a \le x \le b} \left| {\overline{\delta f} \left( {x, \alpha } \right)} \right| < \bar{\varepsilon }} \right)$, then Eq. (46) reduces to

47 $$\left\{ {\begin{array}{*{20}c} { \beta \underline{\delta F}^{\varvec{'}} \left( {x,\alpha } \right) = {\underline{\varepsilon}} + \int\nolimits_{a}^{b} \underline{{k\left( {x,t, \delta F\left( {t,\alpha } \right)} \right)\delta F\varvec{'}\left( {t,\alpha } \right)}} dt } \\ {\beta \overline{{\delta F^{\varvec{'}} }} \left( {x,\alpha } \right) = \bar{\varepsilon } + \int\nolimits_{a}^{b} \overline{{k\left( {x,t, \delta F\left( {t,\alpha } \right)} \right)\delta F\varvec{'}\left( {t,\alpha } \right)}} dt } \\ \end{array} } \right.$$

which can be solved and it confirms the stability of the used numerical approach for solving nonlinear FFIDE-2. In the similar way, stability can also be analysed for nonlinear FVIDE-2.

### Convergence analysis

In this section, we proved that the nonlinear fuzzy Fredholm integro-differential equation of the second kind converges to the exact solution while using the presented approximation technique using HPM. The limit of the solution series as obtained in Eq. (30) is considered as solution of Eq. (15). Assume that ${\underline{F}} \left( {x, \alpha } \right)$ and $\bar{F}\left( {x, \alpha } \right)$ are bounded functions for $a \le x \le b$ and $0 \le \alpha \le 1$.

Let us assume the nonlinear functions in Eqs. (4) and (5) as

48 $$\left\{ {\begin{array}{*{20}c} { \underline{{k\left( {x,t, F\left( {t,\alpha } \right)} \right)F\varvec{'}\left( {t,\alpha } \right)}} = \underline{{k\left( {x,t} \right)G^{r} \left( {t,\alpha } \right)}} } \\ {\overline{{k\left( {x,t, F\left( {t,\alpha } \right)} \right)F\varvec{'}\left( {t,\alpha } \right)}} = \overline{{k\left( {x,t} \right)G^{r} \left( {t,\alpha } \right)}} } \\ \end{array} } \right.$$

#### **Theorem 2**

*The series solution Eq.* (30) *of nonlinear FFIDE-2 as given in Eq.* (15) *using homotopy perturbation method converges to exact solution.*

#### *Proof*

Consider Eqs. (4) and (5) corresponding to Eq. (15) in the form

49 $$\left\{ {\begin{array}{*{20}c} {{\underline{F}}^{\varvec{'}} \left( {x , \alpha } \right) = \underline{f } \left( {x , \alpha } \right) + \lambda \int\nolimits_{a}^{b} \underline{{k\left( {x,t} \right)G^{r} \left( {t,\alpha } \right)}} dt, {\underline{F}} \left( {x_{0} } \right) = \underline{{X_{0} }} _{ } } \\ {\overline{F'} \left( {x , \alpha } \right) = \bar{f} \left( {x , \alpha } \right) + \lambda \int\nolimits_{a}^{b} \overline{{k\left( {x,t} \right)G^{r} \left( {t,\alpha } \right)}} dt, \bar{F} \left( {x_{0} } \right) = \overline{{X_{0} }} } \\ \end{array} } \right.$$

where $$\lambda > 0$$, $$a \le x \le b$$, $$0 \le \alpha \le 1$$.

If the solution series Eq. (30) converges to $\left( {{\underline{F}} \left( {x, \alpha } \right),\bar{F}\left( {x, \alpha } \right)} \right)$, where

50 $$\left\{ {\begin{array}{*{20}c} {{\underline{F}} \left( {x, \alpha } \right) = \int\nolimits_{a}^{b} {\underline{F}}^{\varvec{'}} \left( {x,\alpha } \right)dx} \\ {\bar{F}\left( {x, \alpha } \right) = \int\nolimits_{a}^{b} \overline{{F^{\prime}}} \left( {x,\alpha } \right)dx} \\ \end{array} } \right.$$

Now we can write

51 $$\left\{ {\begin{array}{*{20}c} {{\underline{F}}^{\varvec{'}}_{n} \left( {x , \alpha } \right) = \underline{f } \left( {x , \alpha } \right) + \lambda \int\nolimits_{a}^{b} \underline{{k\left( {x,t} \right)G_{n}^{r} \left( {t,\alpha } \right)}} dt, {\underline{F}} \left( {x_{0} } \right) = \underline{{X_{0} }} _{ } } \\ {\overline{{F^{\prime}}}_{n} \left( {x , \alpha } \right) = \bar{f} \left( {x , \alpha } \right) + \lambda \int\nolimits_{a}^{b} \overline{{k\left( {x,t} \right)G_{n}^{r} \left( {t,\alpha } \right)}} dt, \bar{F} \left( {x_{0} } \right) = \overline{{X_{0} }} } \\ \end{array} } \right.$$

where

52 $$\left\{ {\begin{array}{*{20}c} {\underline{F'} \left( {x , \alpha } \right) = \mathop{lim} \nolimits_{n \to \infty } {\underline{F}}^{\varvec{'}}_{n} \left( {x , \alpha } \right)} \\ {\overline{F'} \left( {x, \alpha } \right) = \mathop{lim} \nolimits_{n \to \infty } \overline{F'}_{n} \left( {x , \alpha } \right)} \\ \end{array} } \right.$$

Subtract Eq. (51) from Eq. (49) correspondingly, we define the error function as

53 $$E_{n} \left( {x , \alpha } \right) = {\underline{E}}_{n} \left( {x , \alpha } \right) + \bar{E}_{n} \left( {x , \alpha } \right)$$

Hence we have

54 $$\left\{ {\begin{array}{*{20}c} {{\underline{E}}_{n} \left( {x , \alpha } \right) = \left( {{\underline{F}}^{\varvec{'}} \left( {x , \alpha } \right) - {\underline{F}}^{\varvec{'}}_{n} \left( {x , \alpha } \right)} \right)\underline{f } \left( {x , \alpha } \right) + \lambda \int\nolimits_{a}^{b} \underline{{k\left( {x,t} \right)G_{n}^{r} \left( {t,\alpha } \right)}} dt } \\ {\bar{E}_{n} \left( {x , \alpha } \right) = \left( {\overline{{F^{\prime}}} \left( {x, \alpha } \right) - \overline{F'}_{n} \left( {x , \alpha } \right)} \right)\bar{f} \left( {x , \alpha } \right) + \lambda \int\nolimits_{a}^{b} \overline{{k\left( {x,t} \right)G_{n}^{r} \left( {t,\alpha } \right)}} dt } \\ \end{array} } \right.$$

At this stage, we have to prove that when $n \to \infty$, the error function $E_{n} \left( {x , \alpha } \right) \to 0$.

Therefore

55 $$\begin{array}{*{20}c} {max} \\ {\forall x \in \left[ {a,b} \right]} \\ \end{array} \left| {E_{n} } \right| = \begin{array}{*{20}c} {max} \\ {\forall x \in \left[ {a,b} \right]} \\ \end{array} \left| {{\underline{E}}_{n} + \bar{E}_{n} } \right|$$

56 $$\begin{aligned} \le \begin{array}{*{20}c} {max} \\ {\forall x \in \left[ {a,b} \right]} \\ \end{array} \left| {{\underline{F}}^{\varvec{'}} \left( {x , \alpha } \right) - F'_{n} \left( {x , \alpha } \right)} \right| + \begin{array}{*{20}c} {max} \\ {\forall x \in \left[ {a,b} \right]} \\ \end{array} \left| {\overline{{F^{\prime}}} \left( {x, \alpha } \right) - \overline{{F^{\prime}}}_{n} \left( {x , \alpha } \right)} \right| \hfill \\ +\begin{array}{*{20}c} {max} \\ {\forall x \in \left[ {a,b} \right]} \\ \end{array} \left| {{\underline{G}}^{r} \left( {t , \alpha } \right) - {\underline{G}}_{n}^{r} \left( {t , \alpha } \right)\left| { + \begin{array}{*{20}c} {max} \\ {\forall x \in \left[ {a,b} \right]} \\ \end{array} } \right|\bar{G}^{r} \left( {t , \alpha } \right) - \bar{G}_{n}^{r} \left( {t , \alpha } \right)} \right| \hfill \\ \end{aligned}$$

57 $$\begin{aligned} \le \left| {\left| {{\underline{F}}^{\varvec{'}} \left( {x , \alpha } \right) - {\underline{F}}^{\varvec{'}}_{n} \left( {x , \alpha } \right)\left| {\left| + \right|} \right|\overline{F'} \left( {x, \alpha } \right) - \overline{F'}_{n} \left( {x , \alpha } \right)} \right|} \right| \hfill \\ \quad + \left| \lambda \right|\int\nolimits_{a}^{b} \left| {\left| k \right|} \right|\left( {\left| {\left| {{\underline{G}}^{r} \left( {t , \alpha } \right) - {\underline{G }}_{n}^{r} \left( {t , \alpha } \right)\left| {\left| + \right|} \right|\bar{G}^{r} \left( {t , \alpha } \right) - \bar{G}_{n}^{r} \left( {t , \alpha } \right)} \right|} \right|} \right)dt \hfill \\ \end{aligned}$$

We know that $\left| {\left| k \right|} \right|$ is bounded. Therefore in the above equation $\left| {\left| {{\underline{G }}^{r} \left( {t , \alpha } \right) - {\underline{G }}_{n}^{r} \left( {t , \alpha } \right)} \right|} \right| \to 0$ and $\left| {\left| {\bar{G}^{r} \left( {t , \alpha } \right) - \bar{G}_{n}^{r} \left( {t , \alpha } \right)} \right|} \right| \to 0$ which implies that $\left| {\left| {E_{n} } \right|} \right| \to 0$. So the series is convergent and the proof is complete. In the similar way, convergence can also be analysed for nonlinear FVIDE-2.

## Numerical illustrations

To illustrate the utility of the technique proposed in this paper, we consider the following examples of nonlinear fuzzy Fredholm and Volterra integro-differential equations.

### *Example 1*

Consider the nonlinear FFIDE-2 given by

$$F^{'\left( x \right)} = \left( {\alpha - \frac{{\alpha^{2} }}{8}, \frac{{12 - 4\alpha - \alpha^{2} }}{8}} \right) + \int\nolimits_{0}^{1} \frac{{t^{2} }}{2}F^{2} \left( t \right)dt$$

$a = 0$, $b = 1$, $\lambda = 1$, $0 \le x$, $t \le 1$, $0 \le \alpha \le 1,$ with the initial conditions ${\underline{F}} \left( {0, \alpha } \right) = \bar{F}\left( {0, \alpha } \right) = 0$

The exact solution of this equation is given by

$${\underline{F}} \left( {x, \alpha } \right) = {\underline{u}} \left( {x,\alpha } \right) = \alpha x$$

$$\bar{F}\left( {x, \alpha } \right) = \bar{u}\left( {x, \alpha } \right) = \left( {2 - \alpha } \right)x$$

Here we have

$$\underline{{K\left( {x,t} \right)}} = \overline{{K\left( {x,t} \right)}} = \frac{t}{2}$$

$${\underline{f}} \left( {x, \alpha } \right) = \alpha - \frac{{\alpha^{2} }}{8}$$

$$\bar{f}\left( {x, \alpha } \right) = \frac{{12 - 4\alpha - \alpha^{2} }}{8}$$

By making use of homotopy perturbation method we may choose a convex homotopy as

$$H\left( {u,p,\alpha } \right) = \left\{ {\begin{array}{*{20}c} {{\underline{u}}^{'} \left( {x,\alpha } \right) - \left( {\alpha - \frac{{\alpha^{2} }}{8}} \right) - p\int\nolimits_{0}^{1} \underline{{F^{2} \left( t \right)}} dt} \\ {\bar{u}^{'} \left( {x,\alpha } \right) - \left( {\frac{{12 - 4\alpha - \alpha^{2} }}{8}} \right) - p\int\nolimits_{0}^{1} \overline{{F^{2} \left( t \right)}} dt} \\ \end{array} } \right.$$

Taking $p^{0} :( {\underline{u}}_{0} \left( {x,\alpha } \right), \bar{u}_{0} \left( {x,\alpha } \right)$) as the initial fuzzy approximations we have

$${\underline{u}}^{\varvec{'}}_{0} \left( {x,\alpha } \right) = {\underline{f}} \left( {x, \alpha } \right) = \alpha - \frac{{\alpha^{2} }}{8}$$

$$\overline{{u^{\prime}}}_{0} \left( {x,\alpha } \right) = \bar{f}\left( {x, \alpha } \right) = \frac{{12 - 4\alpha - \alpha^{2} }}{8}$$

We apply our presented method to approximate the solutions. Hence the HPM series solution will be as follows

$${\underline{u}} \left( {x,\alpha } \right) = \frac{ - 1}{8}\left( { - 8 + \alpha } \right)\alpha + \frac{1}{256}( - 8 + \alpha )^{2} \alpha^{2} - \frac{{( - 8 + \alpha )^{3} \alpha^{3} }}{4096} + \cdots$$

$$\bar{u}\left( {x,\alpha } \right) = \frac{ - 1}{8}\left( { - 2 + \alpha } \right)\left( {6 + \alpha } \right) + \frac{1}{256}( - 12 + 4\alpha + \alpha^{2} )^{2} - \frac{{( - 12 + 4\alpha + \alpha^{2} )^{3} }}{4096} + \cdots$$

**Fig. 1:**
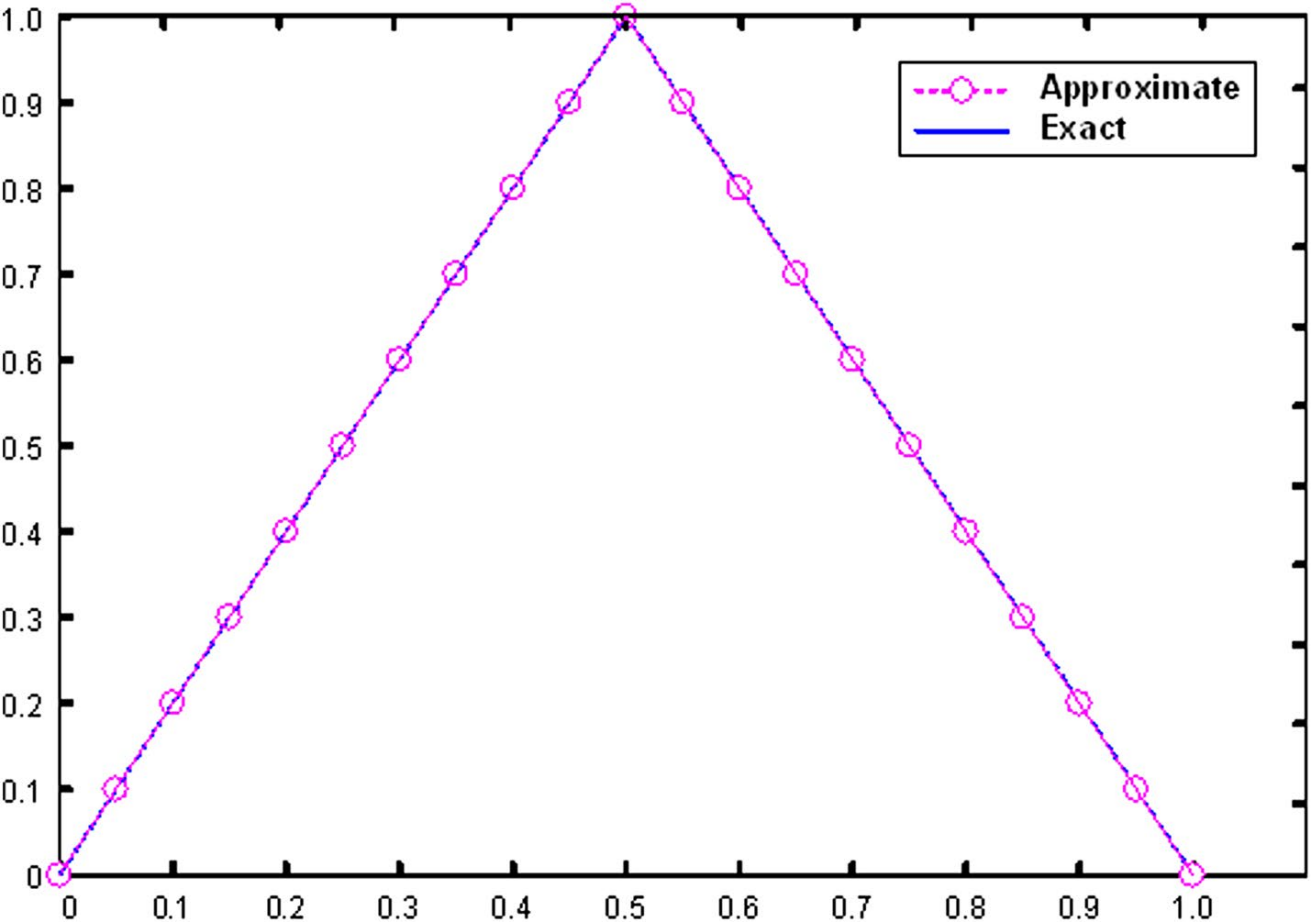
Exact and obtained approximate solutions at *x* = 0.5 for example 1

We solved these equations and found the components of the above iterations by using Mathematica program (Mathematica package version 7). In this case, fuzzy approximate solutions is calculated at four iterations and are given in Table 1. Figure 1 shows the graphical illustration of the obtained approximate solution with the exact solution subject to the initial conditions. We compute the values for $x = 0.5$ and it is noticeable that the approximate solutions are in close proximity to the exact solutions due to the effective convergence of the solution series. In most cases, for the known function series, even the exact solution could be achieved.

**Table 1:** Comparisons between exact and approximate solutions at *x* = 0.5

$\alpha$	Exact solution		Approximate solution		Error	
	${\underline{u}} \left( {x,\alpha } \right)$	$\bar{u}\left( {x, \alpha } \right)$	${\underline{u}} \left( {x,\alpha } \right)$	$\bar{u}\left( {x, \alpha } \right)$	${\underline{E}} \left( {x,\alpha } \right)$	$\bar{E}\left( {x,\alpha } \right)$
0	0	1	0	0.985254	0	0.014746
0.1	0.050000	0.950000	0.048711	0.943658	0.001289	0.006342
0.2	0.100000	0.900000	0.097247	0.896235	0.002753	0.003765
0.3	0.150000	0.850000	0.146107	0.848632	0.003893	0.003680
0.4	0.200000	0.800000	0.197524	0.792325	0.002476	0.007675
0.5	0.250000	0.750000	0.249120	0.749856	0.000880	0.000144
0.6	0.300000	0.700000	0.299771	0.699762	0.000229	0.000238
0.7	0.350000	0.650000	0.3496260	0.649823	0.000374	0.000177
0.8	0.400000	0.600000	0.399712	0.599735	0.000288	0.000265
0.9	0.450000	0.550000	0.449252	0.549753	0.000748	0.000247
1	0.500000	0.500000	0.499783	0.499685	0.000217	0.000315

### *Example 2*

Consider the nonlinear FVIDE-2 given by

$$F^{'\left( x \right)} = \left( {\begin{array}{*{20}c} {\frac{{\alpha^{2} }}{2} + \alpha e^{x} - \frac{{\alpha^{2} }}{2}e^{2x} , } \\ {2e^{x} - \alpha e^{x} + 2 - 2\alpha + \frac{{\alpha^{2} }}{2} - 2e^{2x} + 2\alpha e^{2x} - \frac{{\alpha^{2} }}{2}e^{2x} } \\ \end{array} } \right) + \int\nolimits_{0}^{x} F^{2} \left( t \right)dt$$

$$a = 0$$, $$\lambda = 1$$, $$0 \le t \le x,$$$$0 \le \alpha \le 1,$$with the initial conditions $${\underline{F}} \left( {0, \alpha } \right) = \alpha$$ and $$\bar{F}\left( {0, \alpha } \right) = 2 - \alpha$$.

The exact solution of this equation is given by

$${\underline{F}} \left( {x, \alpha } \right) = {\underline{u}} \left( {x,\alpha } \right) = \alpha e^{x}$$

$$\bar{F}\left( {x, \alpha } \right) = \bar{u}\left( {x, \alpha } \right) = \left( {2 - \alpha } \right)e^{x}$$

Here we have

$$\underline{{K\left( {x,t} \right)}} = \overline{{K\left( {x,t} \right)}} = 1$$

$${\underline{f}} \left( {x, \alpha } \right) = \frac{{\alpha^{2} }}{2} + \alpha e^{x} - \frac{{\alpha^{2} }}{2}e^{2x}$$

$$\bar{f}\left( {x, \alpha } \right) = 2e^{x} - \alpha e^{x} + 2 - 2\alpha + \frac{{\alpha^{2} }}{2} - 2e^{2x} + 2\alpha e^{2x} - \frac{{\alpha^{2} }}{2}e^{2x}$$

We may choose a convex homotopy such that

$$H\left( {u,p,\alpha } \right) = \left\{ {\begin{array}{ll} {{\underline{u}}^{'} \left( {x,\alpha } \right) - \left( {\frac{{\alpha^{2} }}{2} + \alpha e^{x} - \frac{{\alpha^{2} }}{2}e^{2x} } \right) - p\int\nolimits_{0}^{x} \underline{{F^{2} \left( t \right)}} dt} \\ {\bar{u}^{'} \left( {x,\alpha } \right) - \left( {2e^{x} - \alpha e^{x} + 2 - 2\alpha + \frac{{\alpha^{2} }}{2} - 2e^{2x} + 2\alpha e^{2x} - \frac{{\alpha^{2} }}{2}e^{2x} } \right) - p\int\nolimits_{0}^{x} \overline{{F^{2} \left( t \right)}} dt} \\ \end{array} } \right.$$

Taking into account the initial conditions, we have $p^{0} :( {\underline{u}}_{0} \left( {x,\alpha } \right), \bar{u}_{0} \left( {x,\alpha } \right)$) as the initial fuzzy approximations where

$${\underline{u}}^{\varvec{'}}_{0} \left( {x,\alpha } \right) = {\underline{F}} \left( {0, \alpha } \right) = \alpha$$

$$\overline{{u^{\prime}}}_{0} \left( {x,\alpha } \right) = \bar{F}\left( {0, \alpha } \right) = 2 - \alpha$$

Now we begin with the above approximations and applying the proposed numerical technique, we consequently found the HPM series solutions as

$${\underline{u}} \left( {x,\alpha } \right) = \alpha - \alpha x - \frac{{\alpha^{2} }}{2} + \left( {\frac{\alpha }{6} - \frac{{\alpha^{2} }}{3}} \right)x^{3} + \left( {\frac{\alpha }{24} - \frac{{\alpha^{2} }}{6}} \right)x^{4} + \cdots$$

$$\bar{u}\left( {x,\alpha } \right) = 2 - \alpha + \left( {2 - \alpha } \right)x + \left( {1 - \frac{\alpha }{2}} \right)x^{2} + \left( { - 1 + \frac{7\alpha }{6} - \frac{{\alpha^{2} }}{3}} \right)x^{3} + \cdots$$

**Fig. 2:**
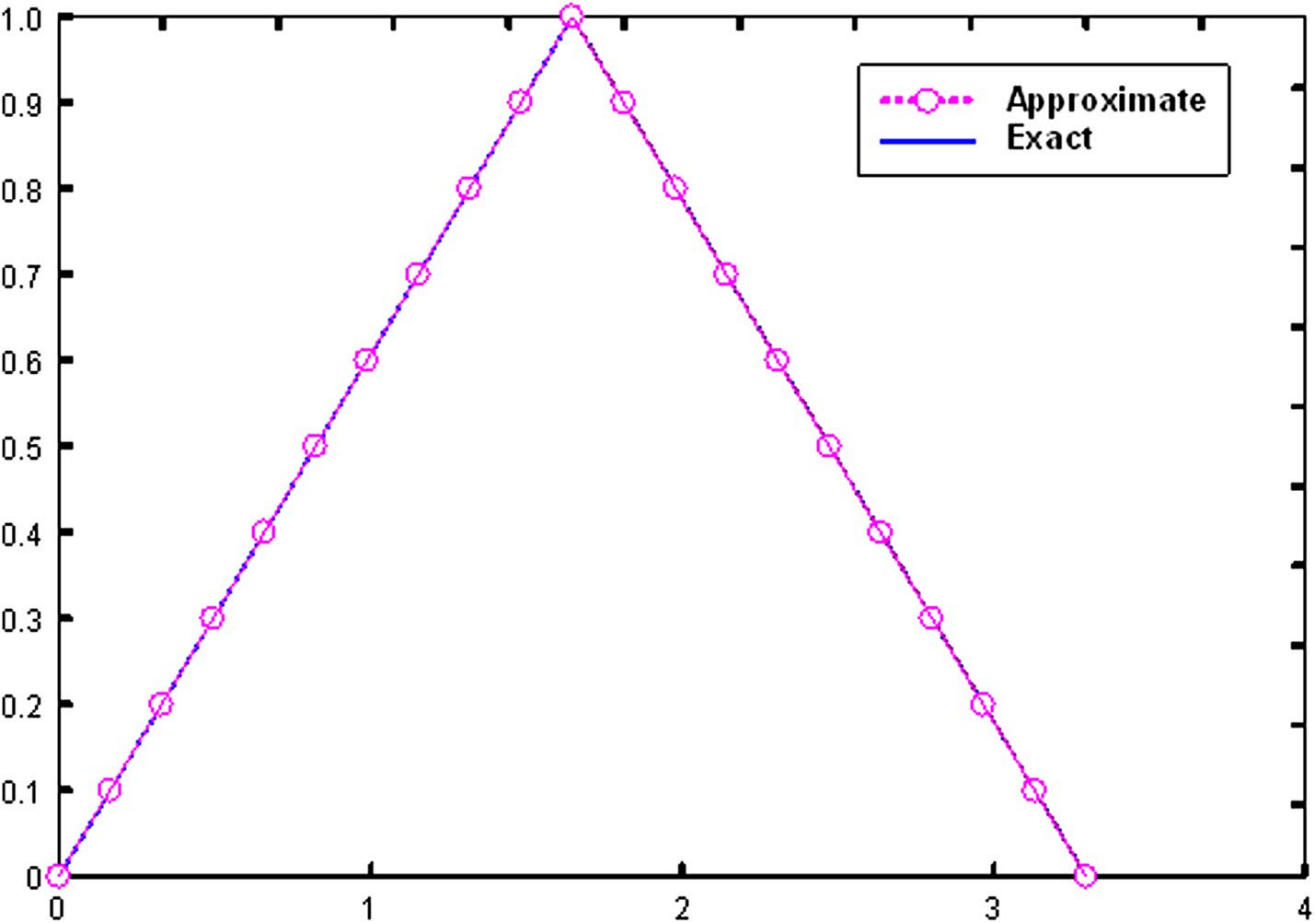
Exact and obtained approximate solutions at *x* = 0.5 for example 2

All the above recursive components were obtained using Mathematica program (Mathematica package version 7). Fuzzy approximate solutions is calculated at four iterations for this example and are given in Table 2. We use $\alpha = 0, 0.1, 0.2, \ldots ,1$ for all fuzzy numbers and calculate the accurate approximations. Besides the graphical representation of exact and approximate solutions for $x = 0.5$ is provided to show the comparison (Fig. 2) and to reveal that the obtained values are nearly accurate to the exact solution.

**Table 2:** Comparisons between exact and approximate solutions at *x* = 0.5

$\alpha$	Exact solution		Approximate solution		Error	
	${\underline{u}} \left( {x,\alpha } \right)$	$\bar{u}\left( {x, \alpha } \right)$	${\underline{u}} \left( {x,\alpha } \right)$	$\bar{u}\left( {x, \alpha } \right)$	${\underline{E}} \left( {x,\alpha } \right)$	$\bar{E}\left( {x,\alpha } \right)$
0	0	3.297440	0	3.297412	0	0.000028
0.1	0.164872	3.132570	0.164718	3.132432	0.000154	0.000138
0.2	0.329744	2.967699	0.329726	2.967489	0.000018	0.000210
0.3	0.494616	2.802826	0.494592	2.802652	0.000024	0.000174
0.4	0.659489	2.637954	0.659456	2.637726	0.000033	0.000228
0.5	0.824631	2.473082	0.824586	2.472886	0.000045	0.000916
0.6	0.989233	2.308210	0.989196	2.307985	0.000037	0.000225
0.7	1.154100	2.143338	1.154069	2.143228	0.000031	0.000011
0.8	1.318980	1.978466	1.318952	1.978395	0.000028	0.000071
0.9	1.483850	1.813593	1.483808	1.813587	0.000042	0.000006
1	1.648720	1.648721	1.648672	1.648706	0.000048	0.000015

## Conclusions

In this paper, we attempt to propose an appropriate approach for solving nonlinear fuzzy integro-differential equations of the second kind by incorporating homotopy perturbation method. Interesting feature of this method is the construction of convex homotopy in a correct way results in precise fuzzy approximate solutions. A detailed proof of the stability analysis and convergence analysis validates effectiveness of the presented method. The methodology has been exemplified by two illustrated numerical examples which prove the computational efficiency. Here the solution is considered as the summation of infinite series which converges rapidly and precision can be improved by taking few more terms in the solution. Numerical results tabulated emphasize the convergence of the solution. This technique can extremely minimize the size of work if compared to existing methods as it reduces the huge calculations needed by them. In fact, this method let to solve in a simpler fashion, the nonlinear fuzzy integro-differential equations which has the advantage in terms of its straightforward selection of the initial approximation and offers reliable accuracy. Future works can be focused on developing a novel method by taking this approach as a basis for solving higher order fuzzy integro-differential equations.With this end, we conclude that the presented method will be a reliable tool to deal with the practical applications of nonlinear fuzzy integro-differential equations.
